# Monitoring the Clinical Response to an Innovative Transdermal Delivery System for Ibuprofen

**DOI:** 10.3390/pharmaceutics11120664

**Published:** 2019-12-09

**Authors:** Anthony Wright, Heather A. E. Benson, Penny Moss, Rob Will

**Affiliations:** 1 School of Physiotherapy and Exercise Science, Curtin University, GPO Box U1987, Perth, WA 6845, Australia; P.Moss@curtin.edu.au; 2 School of Pharmacy and Biomedical Sciences, Curtin University, GPO Box U1987, Perth, WA 6845, Australia; H.Benson@curtin.edu.au; 3 Colin Bayliss Research and Teaching Unit, 27/443 Albany Hwy, Victoria Park, Perth, WA 6100, Australia; robw@bdaus.com.au

**Keywords:** osteoarthritis, magnetophoresis, ibuprofen, transdermal, NSAID, phase 1, clinical evaluation toolkit, transdermal drug delivery systems

## Abstract

We present a phase 1 study that utilizes a crossover design that provides a rapid and relatively inexpensive methodology for evaluating a new transdermal product. The treatment for osteoarthritis (OA) aims to reduce pain and improve function. An innovative magnetophoresis technology has been developed that facilitates transdermal delivery of ibuprofen. The study used measures that were taken over a relatively short time period to monitor the pharmacodynamic response to ibuprofen. Each participant received magnetophoresis-enhanced transdermal ibuprofen or placebo in randomised order, with a five-day washout period. The participants were 24 volunteers with medically diagnosed, painful knee OA. The primary outcome measures were VAS rating of pain on movement and Western Ontario and McMaster Universities (WOMAC) pain and function scores. VAS for pain on movement (*p* < 0.001), WOMAC pain score (*p* = 0.004), and WOMAC function score (*p* = 0.004) were all significantly improved. There was a significant reduction in movement-related pain (*p* < 0.05) during the first patch application and for the remainder of the study period. The number needed to treat for a 50% reduction in movement related pain was 2.2. The study showed a rapid and significant analgesic effect in response to transdermal ibuprofen. A short trial of this nature can be used for informing the parameters that are required for a major randomised controlled trial.

## 1. Introduction

In the International Council for Harmonisation of Technical Requirements for Pharmaceuticals for Human Use (ICH), Harmonised Tripartite Guideline developed in 2008, the committee stated that, in all cases, a pharmaceutical “product should be designed to meet patients’ needs and the intended product performance” [1]. They acknowledged that pharmaceutical development could be achieved through an empirical approach or preferably through a more systematic approach, such as Quality by Design (QbD). In the case of transdermal product development, the ultimate aim is to generate a dosage form that delivers a therapeutic amount of drug to its target site that provides a good clinical outcome with minimal inconvenience and side-effects. The process starts by defining a quality target product profile (QTPP) and the critical quality attributes (CQAs) of the drug product, formulating to those criteria and characterizing the physical, drug release, and delivery properties. Ultimately, the clinical efficacy afforded the patient group to which the product is targeted determines the success of the product development program. Here, we demonstrate how a well-defined set of clinical testing protocols and an efficient clinical trial design can be utilized to demonstrate short term clinical efficacy to achieve proof of concept and facilitate the pharmaceutical product design process in the development of transdermal delivery systems for pain management. We have developed a clinical testing toolkit [2] and short term trial design that allows for the assessment of both pain and function in patients with knee osteoarthritis (OA). This paper describes the toolkit’s application in a short-term, crossover trial to evaluate the key clinical parameters for a novel transdermal patch that delivered a non-steroidal anti-inflammatory drug (NSAID) to painful osteoarthritic knees. This relatively inexpensive phase 1 trial methodology provides an indication of the relative clinical efficacy of the new transdermal product to evaluate the product viability and provide data for underpinning the design of a major randomised controlled trial (RCT).

OA is the most prevalent form of arthritis, causing increasing pain, disability, reduced quality of life, and economic burden worldwide [3,4,5]. Its incidence is directly related to age, with about 50% of the affected individuals aged over 65. The primary objectives of pharmaceutical treatment for OA are to manage pain and improve function, with NSAIDs long being a mainstay of treatment [6]. 

Studies suggest that individuals with knee pain achieve equivalent clinical outcomes whether treated with oral or transdermal NSAIDs over time [7,8,9]. Nevertheless, microdialysis studies indicate that oral administration results in higher tissue levels of ibuprofen in a shorter time-period than transdermal administration [10], and so the immediate onset of effect from transdermal administration is likely to be slower than from oral administration. 

OBJ Ltd. has developed an innovative magnetophoresis technology that facilitates the transdermal delivery of a range of pharmaceutical agents including ibuprofen [11]. Magnetic fields have previously been shown to enhance skin penetration of a range of drugs [12,13,14]. The Kneeguard device has a custom designed ibuprofen hydrogel formulation that is coupled with an OBJ magnetic enhancement backing. It is proposed that the combination of magnetophoresis and optimised hydrogel formulation promotes drug delivery to the target tissues through enhanced partitioning to the skin and diffusion within the skin layers [11]. This device has the potential to facilitate more rapid absorption of ibuprofen and, hence, ensure that transdermal administration can achieve good clinical outcomes (pain relief and improved function) over a short time period. 

The aim of the study was to provide initial evidence of clinical outcomes following 48 h administration of magnetophoresis-enhanced transdermal ibuprofen (5%), in comparison to placebo.

The primary objective was to determine whether magnetophoresis-enhanced transdermal delivery of ibuprofen achieved superior outcomes to placebo over 48 h. A secondary objective was to provide information about number needed to treat (NNT), effect size, time to onset of effect, and duration of effect as a means of comparing with existing products to provide an indication of product effectiveness and assist in designing a major clinical trial, as the next step in the product development process. In addition, the trial provided information regarding any potential adverse events to inform a future clinical trial.

## 2. Materials and Methods

The study utilized a double-blind, repeated measures design to evaluate the intervention over a relatively short (48 h) time-period. Each participant completed two study periods in which they received either magnetophoresis-enhanced transdermal ibuprofen patches or placebo patches in randomised order. There was a minimum five-day washout period between each study period. Utilizing a crossover design at this early stage in the development process has advantages over the conventional parallel group design. Each participant acts as their own control, which minimizes confounding variables and the design is advantageous in improving the power of the statistical tests used to evaluate the treatment effect, which results in a smaller sample size being required [15].

### 2.1. Participants

The participants were community-dwelling volunteers with painful knee OA that were recruited via internet advertisement and diagnosed with OA by a Rheumatologist. The exclusion criteria included: history of neurological disorders affecting sensory, motor or cognitive function; recent lower limb injury or surgery; history of gastric ulcers or renal disease; known or suspected allergy/sensitivity to ibuprofen; known or suspected allergy/sensitivity to adhesive or tape; and, history of any serious adverse events related to previous NSAID use. The study was conducted with a pre-determined sample size of 24 participants at the Curtin University Health and Wellness Centre. All of the participants provided written informed consent and the Human Research Ethics committee at Curtin University provided ethical approval (HRE2017-0528, 5 October 2017). The trial was registered in the Australia and New Zealand Clinical Trials Registry (ACTRN12617000841370): full details of the trial design are available from the ANZCTR website.

### 2.2. Medications

All of the active and placebo patches were managed and stored in the School of Pharmacy and Biomedical Sciences at Curtin University. Each participant received identical packs, with three days’ supply of Kneeguard patches (OBJ Ltd, Leederville, Australia) containing 5% ibuprofen hydrogel (125 mg) or identical placebo patches containing hydrogel with no active constituent. The gel was formulated using a hydroalcoholic solvent base consisting of isopropyl alcohol, propylene glycol, and water, which was optimized based on the release of ibuprofen (using standard in house in vitro release test: IVRT) and minimizing skin irritation. The order in which participants received the active patch or the placebo patch was randomised, with both participants and investigator blind to the order. The participants completed a washout period equal to five half-lives of their usual analgesic or anti-inflammatory medication before baseline testing, for each study period. They were permitted to use paracetamol as rescue mediation during the washout period if required, but they were not permitted to use any additional analgesic or anti-inflammatory medication during the study periods. Individual patches were worn for a period of five hours, followed by a 30-minute rest interval before a new patch was applied. Patches were not worn while the participants were in bed at night. Testing was carried out over a three-day period.

The study Statistician produced a computer generated randomisation schedule and the patches were allocated by the study Pharmacist based on that schedule. Allocation was counterbalanced, such that each experimental condition (active/placebo) was administered to an equivalent number of participants in each study period. A new participant replaced participants who failed to complete both study periods. 

### 2.3. Self-Report Measures

The ratings of resting pain and pain on movement (pain during moving from sitting to standing up from a standard dining chair three times) [16] were obtained while using visual analogue scale (VAS) ratings. These ratings were taken at baseline, at the end of each 48 h study period, and also by participants at home every 2 to 2.5 h during the patch application periods. An iPad app provided an alarm to alert participants to the need to complete these ratings. The participants rated their resting pain and pain during sitting to standing on a 10 cm VAS, with end points marked “no pain” and “worst pain imaginable”. The iPad app also provided them with alerts regarding when to change their Kneeguard patches. Pain, stiffness, and dysfunction were assessed at baseline and at the end of each 48 h study period using the Western Ontario and McMaster Universities (WOMAC) Osteoarthritis Knee Index. This questionnaire has been widely used to measure pain and disability in OA, demonstrating good internal validity and test-retest reliability [17]. 

### 2.4. Physical Function Tests

Physical function was assessed at baseline and at the end of the 48 h study period using the three tasks of the Aggregated Locomotor Function Score (ALF) [2,18] in standardized order. The time taken to complete the three tasks was measured using a stop watch: stand-to-sit-to-stand (walk two metres to a chair, sit, stand, and return); eight metre return walk; and, stairs (14 step ascent and descent). Each task was completed three times and the mean time (seconds) calculated. Pain during each locomotion task was also assessed using a 10 cm VAS, with endpoints marked “no pain” and “worst pain imaginable”.

### 2.5. Quantitative Sensory Test

Pressure pain threshold (PPT) was assessed while using an electronic digital pressure algometer (Somedic AB, Sosdala, Sweden) [19] at the medial joint line of the affected knee. The 1 cm^2^ algometer probe was applied at 90° to the skin at a rate of 40 kPa/s. The participants were instructed to depress the hand-held switch as soon as the sensation of pressure became painful [20]. Lower PPT values indicate greater sensitivity. The PPT tests were carried out in triplicate and the mean pressure value (kPa) calculated. This measure was completed at baseline and at the end of each 48-hour study period. An investigator who was blinded to treatment allocations throughout the study carried out all of the testing.

### 2.6. Global Rating of Change

At the 48-hour follow-up, participants completed a five-point global rating of change (GROC) score (“Since wearing the patch, do you think that your pain is: much worse, worse, the same, better or much better than usual”) [21].

### 2.7. Procedure

All of the participants completed testing in a standardised order. The completion of the WOMAC questionnaire was followed by VAS ratings of pain at rest and pain on movement (repeated sit-to-stand X 3). ALF functional tests were followed by an assessment of PPT at the affected knee. After completion of the test battery, the participants were provided with a medication package containing a supply of Kneeguard adhesive patches (5% ibuprofen) or placebo patches for that particular study period. The participants with bilateral knee OA were provided with sufficient patches to use for both knees. They were instructed on how to apply the patches, how to record their VAS ratings, and when to replace the patches. An illustrated instruction sheet was included with each medication package. The first patches were applied before the participant left the test session. The participants received a follow-up text after 24 h to ensure that they were following the protocol and had not experienced any difficulties with the test procedures or any adverse events related to the patches.

All of the participants returned to the research laboratory two days later and completed the same battery of tests in the same order of testing. They were also asked to record a global rating of change in knee pain since wearing the patches and were questioned about any adverse events that they had experienced over the prior 48 h. They were asked to return any unused patches. 

The same procedure was followed in a second study period, at least one week later, when the participants received the alternative patch (Ibuprofen or placebo). The participants were permitted to use their normal medications during the intervals between testing, but they were required to complete a medication wash-out before the commencement of each study period.

### 2.8. Adverse Event Recording

The participants received a text to inquire about adverse events after 24 h and they were also questioned regarding adverse events at the 48-hour assessment. Any adverse events were fully documented, indicating any likely association with the study medications, the patch adhesive or the study procedures. Skin irritation ratings were categorized on a four-point scale according to severity, with 3 indicating skin breakdown (e.g., blistering, raw, breakdown of the skin etc.), 2 indicating minor changes in skin condition (e.g., dry, flaky, red or rash), and 1 indicating symptoms of altered sensation (e.g., itchy, heat, tingling) and 0 indicating no reaction at all [21].

### 2.9. Data Analysis

The primary outcome measures were VAS rating of pain on movement, WOMAC pain score, and WOMAC function score. The secondary outcome measures included VAS rating of pain at rest, WOMAC stiffness, ALF score, ALF pain rating, and PPT. Analysis was conducted on a per protocol basis. The participants were included in the analysis if they had used all of the patches, completed baseline and 48-hour testing for each study period, and the majority of VAS ratings (8/10). The participants who failed to complete pre and post testing for both study periods were replaced and their data were not included in the analysis.

Data were analysed while using a two-factor repeated measures ANOVA with treatment condition (Active/Placebo) and time (Pre/Post, or multiple time points) as the independent variables. For analyses involving multiple time-points (VAS measures), if there was a significant interaction effect paired t-tests were applied at each time point to determine the time periods at which there was a significant difference between the treatment conditions. The effect sizes (Cohen’s d) were calculated for the primary outcome measures and NNT values for a 50% reduction in movement and resting VAS values were calculated to provide information to support a future RCT and as a basis for comparison with other published studies.

## 3. Results

**Figure 1:**
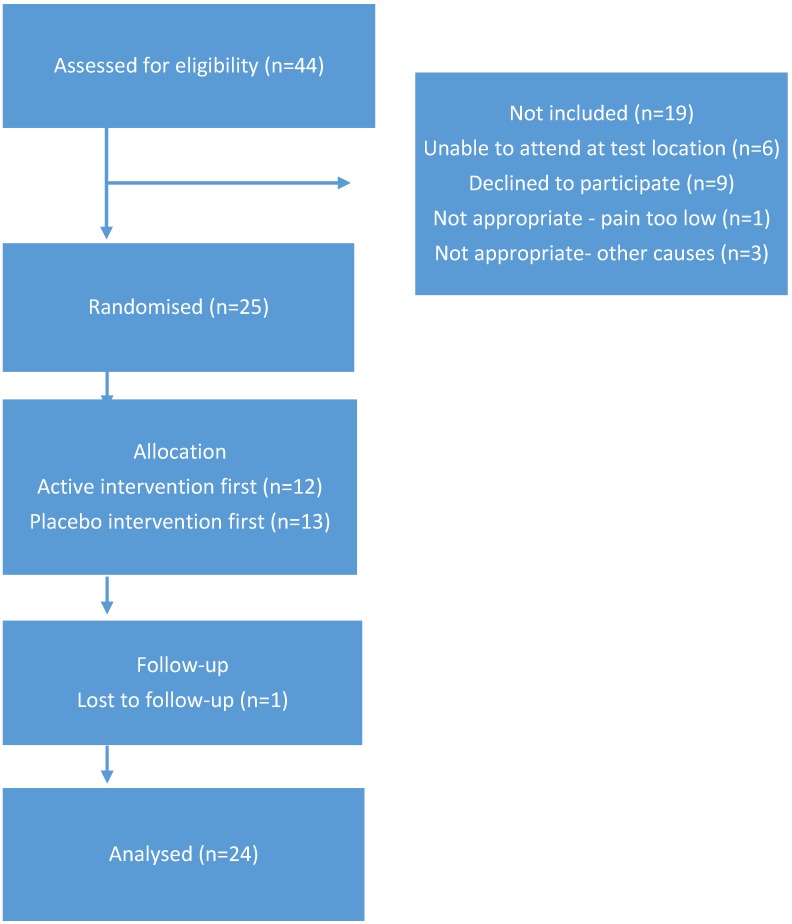
CONSORT diagram indicating participant progression in the study.

Twenty-four participants (six male: 18 female, mean age 66, range 60–77) with medically diagnosed painful knee OA completed the study. The cohort had a mean BMI of 30.9 with five classified as normal weight, eight as overweight, and 11 classified as obese. Whilst there were a range of BMI values, this would have minimal impact on study outcome, as each participant acted as their own control. Therefore, randomisation was not stratified based on BMI score. Table 1 includes further information regarding the cohort. Forty-four individuals expressed interest in the project and completed telephone screening. Twenty-five participants completed consent documentation and commenced the study. One participant withdrew due to difficulties in attending the test venue at the required times and was replaced by an additional participant. Figure 1 illustrates the CONSORT diagram outlining participant numbers.

### 3.1. Primary Outcome Measures

**Figure 2:**
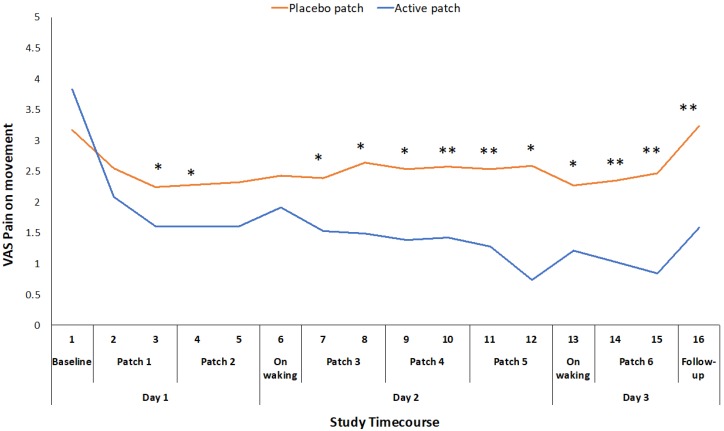
Difference in pain on movement VAS ratings between placebo and active patches for each measurement time-point from baseline to 48-hour follow-up. * significant *p* < 0.05; ** significant *p* < 0.01.

The primary outcome measures of VAS for pain on movement (F_1,46_ = 30.074, *p* < 0.001), WOMAC pain score (F_1,46_ = 9.005, *p* = 0.004), and WOMAC function score (F_1,46_ = 9.838, *p* = 0.003) were all significantly different between the active patch session and the placebo patch session (Table 2), with the participants experiencing less pain and improved function during the active patch session. VAS for pain on movement was recorded at 16 time points throughout the 48-hour study periods. There was a significant treatment*time interaction effect for this measure (F_1,46_ = 8.864, *p* < 0.001), and there was a significant difference between the active and placebo patch sessions during the initial patch application and consistently from the initial evaluation on day 2 until the completion of the study (Figure 2).

The effect sizes were calculated for all of the primary outcome measures (Table 3), and indicated effect sizes for ibuprofen relative to placebo in the small to moderate range.

### 3.2. Secondary Outcome Measures

**Figure 3:**
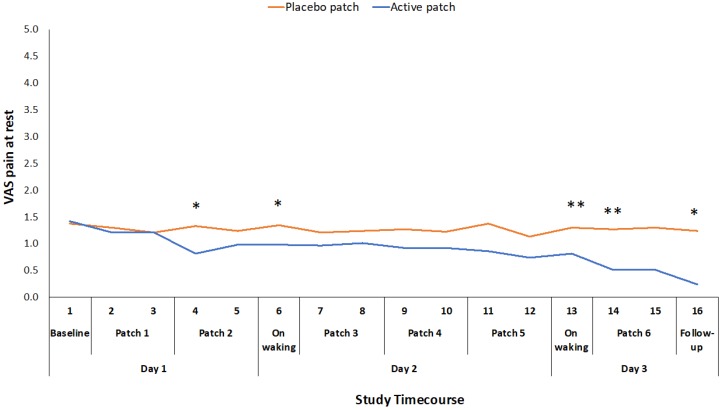
Difference in pain at rest VAS ratings between placebo and active patches for each measurement time-point baseline to 48-hour follow-up. * significant *p* < 0.05; ** significant *p* < 0.01.

Of the secondary outcome measures that were related to pain and function, VAS for pain at rest, WOMAC stiffness score, and PPT were all significantly improved for the active patch session when compared to the placebo patch session (Table 2). There was no significant difference between the sessions for the total time that was required to complete the three movement tasks in the ALF test (Table 2), although there was a significant reduction in the time taken to complete the walk component of the test and the time taken to complete the stairs component approached significance. There was also a significant difference in participants’ rating of pain during the three component tests (sit-to-stand, walk and stairs) of the ALF, with the participants experiencing less pain during these functional tasks following the active patch session. VAS for pain at rest was also recorded at 16 time points throughout the 48-hour study period. There was a significant treatment*time interaction effect for this measure (F_1,46_ = 2.416, *p* = 0.017), although the difference was only significant at intermittent time points through the 48-hour study period (Figure 3).

The NNT for a 50% reduction in VAS values indicated that the NNT for pain on movement was 2.2 and the NNT for pain at rest was 3.4.

### 3.3. Global Rating of Change

At the 48-hour follow-up, 22 participants indicated that their pain was better or much better following the active patch application, with only one participant indicating that their pain was better following the placebo patch application (Table 4).

### 3.4. Patch Usability, Adverse Events and Skin Irritation

The Kneeguard patches remained in place on the knee site and permitted a normal range of motion and physical activity. There were no major adverse events recorded during the study. The participants were asked to check their skin every time they removed a patch. Overall, the patches were well tolerated. The majority of participants (75%—18/24) reported no skin reactions to either placebo or active patches. Five participants with the active patches reported light skin redness and six participants with the placebo patches (Table 5). In all cases, this was experienced in the area of the adhesive tape and not the gel. One participant reported an itchy sensation that lasted for several hours on application of the first active patch, but this did not reoccur with subsequent patches.

## 4. Discussion

The Kneeguard patch containing ibuprofen (5%) produced a significantly greater reduction in pain and improvement in function than the placebo patch over a relatively short time period during this cross-over trial. This was particularly the case for movement-related pain. The reduction in pain was apparent with both VAS pain ratings and WOMAC pain score. There was also a clear improvement in function, based on change in WOMAC function score. There was no significant reduction in the total time that was required to complete a series of three functional tasks (ALF), although there was a significant reduction in the time for the walk task and a significant reduction in the level of pain that participants experienced while completing each of the three component tasks. The anti-inflammatory effect of ibuprofen might also be linked to the improvement in stiffness (reduced WOMAC stiffness score) that was reported by many participants and the reduction in pressure sensitivity (tenderness), as indicated by the significant improvement in pressure pain thresholds.

The mean reduction in VAS pain on movement (standing from a chair three times) during the active treatment period of 22.5 mm exceeds the established minimum clinically important difference (MCID) value for a VAS measure of pain on movement in the knee OA population (19.9 mm) [22]. Changes in WOMAC pain and function scores also exceeded the reported MCID values for those measures [23]. This suggests that the topical administration of ibuprofen, utilizing the Kneeguard patch, achieves a clinically important improvement in pain within a 48-hour period. The reduction in movement-related pain was apparent early on day 2 and it was maintained throughout the remainder of the 48 h test period (Figure 2). The reduction in pain at rest was more variable and statistically significant at intermittent points over the 48-hour period (Figure 3).

There are a limited number of studies that have evaluated the effects of topical ibuprofen on pain and function in OA sufferers over short time periods. Tiso and colleagues evaluated topical and oral ibuprofen over a two-week period in people with chronic knee pain [8]. Following one week of topical ibuprofen application, they reported reductions in pain, stiffness, and function on the WOMAC questionnaire of 7%, 16% and 14.4%, respectively. The comparable reductions in pain, stiffness, and function during the current study after 48 hours were 59.5%, 56%, and 46.3%, which suggests that the Kneeguard delivery system achieved superior results when compared with a standard topical application. This supports the company’s internal pharmaceutical product development data including in vitro skin permeation data showing the enhanced delivery of drugs when applied with the magnetophoresis enhancement technology [11].

In a small pilot study, Arendt-Nielsen and colleagues [24] evaluated the effect of a topical ibuprofen cream in comparison to placebo cream applied to the hands in patients with rheumatoid arthritis. A follow-up evaluation occurred after three and seven days. VAS rating of pain on movement showed a reduction of 4.2 mm after three days and 15.5 mm after seven days, in comparison to the 22.5 mm in the current study after 48 h. They also evaluated change in pressure pain threshold at the finger joints, reporting an increase in PPT of 10 kPa after three days and an increase in PPT of 17 kPa after seven days. By comparison, the increase in PPT in the present study was 64.19 kPa after 48 h. It should be acknowledged that this was a different test site, but it does suggest that the treatment that was applied in the current study was more effective in reducing pain sensitization than the topical ibuprofen cream. However, it should be noted that this increase in PPT did not reach the MCID of 114 kPa [25], and so a longer period of treatment might be required to achieve a clinically important level of improvement in sensitivity (tenderness).

Topical ibuprofen gel was compared to oral ibuprofen in a study evaluating the influence on painful soft tissue injuries [9]. The primary outcome was time that was required to become completely better and there was no significant difference between the treatment groups in achieving that outcome (active gel 14 days, active tablet 13.5 days). Pain at rest decreased by 6 mm in the active gel group and 5 mm in the active tablet group after three days, and there was no significant difference between the oral or topical treatments. It is difficult to make any direct comparisons between this study and the current study, because of the differences in methodology, but the study by Whitefield and colleagues does suggest that topical ibuprofen treatments can achieve comparable outcomes to oral ibuprofen.

There are a limited number of published studies with which to compare these data, particularly in relation to topical ibuprofen. A major Cochrane systematic review evaluated topical NSAIDs for the treatment of chronic musculoskeletal pain in adults [26]. The majority of the reviewed studies evaluated topical NSAIDs in the treatment of knee osteoarthritis. The authors considered that diclofenac and ketoprofen were the only NSAIDs with sufficient good quality studies for meta-analysis to be carried out. They calculated the NNT for a 50% reduction in pain rating in order to compare between studies. For diclofenac studies with a 6–12 weeks follow-up, the active to placebo NNT was 9.8 (7.1–16). For studies lasting 2–6 weeks the NNT was 5.0 (3.7–7.4). For ketoprofen studies with a 6–12 weeks follow-up, the NNT was 6.9 (5.4–9.3). As a point of comparison, the NNT for movement pain in the current study was 2.2 and the NNT for pain at rest was 3.4. These values suggest a superior clinical effect of the magnetophoresis enhanced ibuprofen delivery.

The Cochrane report included one recent study evaluating topical ibuprofen in comparison to placebo and while using similar outcome measures to the current study (pain at reset, pain on movement, WOMAC pain, and WOMAC function) [27]. The trial evaluated a novel drug delivery formulation, termed a vasoactive and lipid encapsulated (VALE) cream. The treatment was evaluated at one-week and two-week follow-up assessments. The effect sizes that were calculated for the Varadi study were: pain on movement 0.109, pain at rest 0.219, WOMAC pain 0.089 and WOMAC function 0.095. This compares with effects sizes of 0.675, 0.434, and 0.331, respectively, for the current study (Table 3), which indicated superiority for the ibuprofen magnetophoresis device on all measures. 

Conducting a short phase 1 study, utilizing a crossover design, with an appropriate toolkit of measures provided a rapid and relatively inexpensive methodology to evaluate a new transdermal product, to obtain an indication of its relative clinical efficacy and demonstrate proof of concept. The crossover design increases the statistical power and reduces the required sample size [15]. In addition, focusing on measures, such as effect size, NNT, and MCID, provides a means to compare the efficacy of the product to existing treatments and ensures that the trial outcome is not wholly dependent on demonstrating a statistically significant difference in circumstances where a small sample size might increase the risk of Type II error. The patch applications were carefully monitored for any adverse events, particularly skin irritation. Participants’ experiences with the patches were very positive and skin irritation was relatively minor. Having data on adverse events and product safety provides the opportunity to make some modifications to formulation and product design if these are required prior to conducting a major RCT.

## 5. Conclusions

The current study evaluated the effect of a Knee guard wearable patch, incorporating magnetophoresis to facilitate the transdermal delivery of ibuprofen (5%), in comparison to a placebo patch in people with knee osteoarthritis. Although the study was conducted over a relatively short timeframe, there were significant differences between the active and placebo treatments for all of the primary outcomes. This suggests that the ibuprofen patch produces a significant reduction in pain, particularly movement related pain, and a significant improvement in perceived function, even after 48 h. A number of indicators (effect size, NNT, MCID) suggest that the treatment has a meaningful clinical effect, which would encourage further product development. The findings of this early stage study, while using an efficient study design and relevant assessment toolkit, suggest that it would be beneficial to conduct further research to evaluate the effects of the treatment over longer time periods in patients with knee OA.

## Figures and Tables

**Table 1:** Participant baseline information.

Characteristics	Measure/Number	
**Gender**	**Male: 6**	**Female: 18**
Age: mean (range)	66.0 (60–77) years	
BMI: mean (range)	30.9 (23.2–42.4)	
BMI category (n):	Normal	5
	Overweight	8
	Obese	11
**Osteoarthritic (index) knee**	**Right: 11**	**Left: 13**
Pain in index knee: mean (SD)	5.63 (1.66)	
Pain in non-index knee: mean (SD)	2.92 (2.48)	
Bilateral/unilateral OA knee (n)	Unilateral: 7	Bilateral: 17

**Table 2:** Pre and post values for primary and secondary outcome measures for active and placebo sessions.

Measure	Placebo Pre	SD	Placebo Post	SD	Active Pre	SD	Active Post	SD	N	F_1,46_	*P*
Primary outcome measures											
VAS Move	31.67	26.85	32.29	28.36	38.33	25.78	15.83	19.60	24	30.07	<0.001
WOMAC Pain	112.50	92.51	97.25	108.77	142.04	119.25	57.54	69.882	24	9.005	0.004
WOMAC Function	421.63	367.1	377.04	383.05	480.29	396.30	258.12	334.51	24	9.838	0.003
Secondary outcome measures											
VAS Rest	13.75	24.42	12.29	24.32	14.17	21.09	2.29	6.59	24	4.620	0.037
WOMAC Stiffness	75.33	47.07	58.25	49.78	83.75	51.75	36.83	38.764	24	7.571	0.008
PPT	269.96	89.67	235.62	94.77	253.90	93.58	318.10	121.98	24	36.37	<0.001
ALF Total Time	35.264	8.73	35.827	8.32	36.384	8.61	35.55	10.25	22	0.826	0.368
ALF STS Time	7.21	2.76	7.32	2.99	7.73	3.95	7.88	5.56	24	0.001	0.972
ALF Walk Time	15.22	4.69	15.66	4.92	15.89	6.07	14.42	3.57	24	6.813	0.012
ALF Stairs Time	14.69	5.20	14.91	5.07	15.52	5.66	14.30	5.02	22	3.794	0.058
ALF STS VAS	27.29	30.25	27.71	27.50	35.21	25.52	13.54	20.56	24	16.54	<0.001
ALF Walk VAS	22.92	25.32	21.87	24.79	28.54	25.17	14.79	20.46	24	10.35	0.002
ALF Stairs VAS	36.82	22.71	38.18	23.83	44.09	22.23	23.63	23.1	22	2.90	<0.001

**Table 3:** Effect size (Cohen’s d) calculations for the ibuprofen patch relative to the placebo patch.

Measure	Cohen’s d
Pain on movement	0.675
WOMAC pain	0.434
WOMAC function	0.331

**Table 4:** Global rating of change following either placebo or active patch application.

	Active Patch	Placebo Patch
Much better	12	0
Better	10	1
Same	2	19
Worse	0	3
Much worse	0	1

**Table 5:** Skin reaction to patch applications.

Degree of Skin Reaction	0: None	1: Altered Sensation e.g., Itchy	2: Minor Visible Skin Changes e.g., Redness	3: Major Skin Changes e.g., Skin Breakdown
Placebo patches	18	0	6	0
Active patches	18	1	5	0
